# Assessment of patient-ventilator breath contribution during neurally adjusted ventilatory assist in patients with acute respiratory failure

**DOI:** 10.1186/s13054-015-0775-2

**Published:** 2015-02-18

**Authors:** Ling Liu, Songqiao Liu, Jianfeng Xie, Yi Yang, Arthur S Slutsky, Jennifer Beck, Christer Sinderby, Haibo Qiu

**Affiliations:** Department of Critical Care Medicine, Zhongda Hospital, Southeast University, School of Medicine, 87 Dingjiaqiao Street, Nanjing, 210009 China; Keenan Research Centre for Biomedical Science and Department of Critical Care, St Michael’s Hospital, 30 Bond Street, Toronto, ON M5B1W8 Canada; Department of Medicine and Interdepartmental Division of Critical Care Medicine, University of Toronto, Suit RFE3-805, 200 Elizabeth Street, Toronto, ON M5G 2C4 Canada; Department of Pediatrics, University of Toronto, 555 University Avenue, Toronto, ON M5G 1X8 Canada

## Abstract

**Introduction:**

We previously showed in animals that the ratio of inspired tidal volume (Vt_insp_) to inspiratory peak electrical activity of the diaphragm (EAdi_pk_) can be used to quantify the respective patient and ventilator breath contributions (PVBCs) during neurally adjusted ventilatory assist (NAVA). The PVBC index has not been tested clinically.

**Methods:**

We studied 12 intubated and mechanically ventilated patients with acute respiratory failure and measured EAdi_pk_, airway (Paw) and inspiratory esophageal pressure (Pes) and Vt_insp_. We applied 11 different NAVA levels, increasing them every 3 minutes in steps of 0.3 cm H_2_O/μV from 0 to 3.0 cmH_2_O/μV. At each NAVA level, one breath was non-assisted (NAVA level 0). PVBC indices were calculated by relating Vt_insp_/EAdi_pk_ of the non-assisted breath to Vt_insp_/EAdi_pk_ of the assisted breath(s) using one (^N1^PVBC) or the mean value of five preceding assisted breaths (^X5^PVBC). During assisted breaths, inspiratory changes in Pes (∆Pes) and transpulmonary (ΔPtp) pressures were used to calculate the relative contribution of patient to total inspiratory lung-distending pressures (ΔPes/ΔPtp). Matching of respiratory drive indices and squaring of the PVBC was evaluated for their effect on the correlation between PVBC and ΔPes/ΔPtp. Linear regression analysis and Bland-Altman analysis were applied to compare indices.

**Results:**

Using an average of five assisted breaths prior to the non-assisted breath and squaring the PVBC (^X5^PVBC^2^) improved determination coefficients (*P* <0.05), adjusted the regression slope and intercept between PVBC and ΔPes/ΔPtp toward identity (*P* <0.05) and reduced bias (*P* <0.05). Matching EAdi_pk_ between non-assisted and assisted breaths within the range of 0.77 to 1.30 improved the relationship between ^X5^PVBC^2^ and ΔPes/ΔPtp (*P* <0.05) and abolished the need for EAdi normalization in the PVBC calculation (*R*^2^ = 0.96; bias = 0.16 ± 0.06; precision = 0.33 ± 0.08 (mean and 95% confidence interval)).

**Conclusions:**

This clinical study confirms previous experimental results showing that the PVBC^2^ predicts the contribution of the inspiratory muscles versus that of the ventilator during NAVA, when differences in effort (EAdi) between non-assisted and assisted breaths are limited. PVBC could help to quantify and standardize the adjustment of the level of assist, and hence reduce the risks of excessive ventilatory assist in patients.

**Trial registration:**

ClinicalTrials.gov NCT01663480. Registered 9 August 2012.

## Introduction

The aim of today’s approach to mechanical ventilation is generally to achieve more spontaneous breathing and greater use of so-called partial ventilatory assist, whereby the ventilator and the patient share the inspiratory work. In order for partial ventilatory assist to be efficient in patients who are spontaneously breathing, it must be synchronized in terms of both its timing and its magnitude. However, conventional modes of partial ventilatory assist are often associated with patient–ventilator asynchrony or dyssynchrony [1-3]. Neurally adjusted ventilatory assist (NAVA) is a mode that uses the electrical activity of the diaphragm (EAdi) to synchronize ventilation, and it has been demonstrated to fulfill these requirements [4-11].

One of the biggest unknowns with the use of partial ventilatory assist is the relative contribution of patient effort and ventilator assistance during a breath. An index for quantifying the respective patient and ventilator breath contributions (PVBCs) using an experimental model of acute respiratory failure (ARF) in animals ventilated with NAVA was recently published [12]. In the PVBC index, the ratio of inspiratory tidal volume (Vt_insp_) and peak inspiratory electrical activity of the diaphragm (EAdi_pk_) was used for both an assisted breath and a subsequent breath with no assistance [12].

To validate the PVBC index in our previous study [12], we measured esophageal pressure (Pes) as reflecting pleural pressure and representing the patient’s inspiratory effort. After measuring the airway pressure (Paw) delivered by the ventilator, we calculated the transpulmonary pressure (Ptp) as Paw − Pes to quantify the total pressure applied to distend the lungs. The ratio of inspiratory changes in Pes and Ptp (ΔPes/ΔPtp) was used to represent the fraction/contribution of esophageal pressure (patient) to total inspiratory pressure (patient + ventilator) during NAVA. In our previous experimental study [12], the PVBC index was found to correlate closely to ΔPes/ΔPtp, and it was found that squaring of the PVBC produced a near-perfect linear relationship.

On the basis of the findings of our previous experimental study, we designed the present study to evaluate the PVBC index in humans with ARF. Similar to our previous work in animals [12], the underlying hypothesis was that a simple measurement of Vt_insp_ and EAdi_pk_ between a non-assisted breath and the preceding ventilator-assisted breath(s) would allow the calculation of the PVBC index in humans. We carried out the study with the following assumptions. (1) EAdi_pk_ during an inspiration reflects neural demand to generate Vt_insp_. (2) If adding synchronized assistance to the patient’s neural inspiratory demand, the resulting Vt_insp_ depends on the sum of patient and ventilator pressure contribution. (3) If the neural inspiratory demands are different for two consecutive breaths, normalizing the Vt_insp_ to EAdi_pk_ (Vt_insp_/EAdi_pk_, in units of l/μV), a PVBC index can be constructed using the ratio of Vt_insp_/EAdi_pk_ without ventilatory assistance to Vt_insp_/EAdi_pk_ with assistance (that is, (Vt_insp_/EAdi_pk_)_no-assist_/(Vt_insp_/ΔEAdi_pk_)_assist_); a PVBC index close to 1 suggests that Vt_insp_ is generated by the patient, whereas a PVBC index close to 0 indicates that Vt_insp_ is generated by the ventilator. (5) If the patient’s neural inspiratory demand is similar during both non-assisted and assisted breaths, the ratio of Vt_insp_ alone during the non-assisted and assisted breaths should reflect the relative contribution of the patient vis-à-vis patient + ventilator.

If reliable, PVBC could help to quantify and standardize the adjustment of the level of ventilatory assistance and reduce the risks of excessive ventilatory assistance in patients.

## Material and methods

This trial was conducted in a 30-bed general intensive care unit (ICU) of a teaching hospital affiliated with Southeast University in China. The protocol was approved by the Institutional Ethics Committee of Zhongda Hospital (approval number 2010ZDLL018.0), and informed consent was obtained from the patients or their next of kin. The trial is registered at ClinicalTrials.gov (NCT01663480).

### Patients

Patients were eligible for inclusion if they were (1) intubated or tracheotomized, (2) undergoing assisted mechanical ventilation, (3) considered (by the attending physician) to be able to tolerate a short period of spontaneous breathing (not having passed spontaneous breathing trial prior to study) and (4) awake.

The exclusion criteria were (1) age <18 years; (2) history of esophageal varices; (3) gastroesophageal surgery in the previous 12 months or gastroesophageal bleeding in the previous 30 days; (4) coagulation disorders (international normalized ratio >1.5 and activated partial thromboplastin time >44 seconds); (5) history of acute central or peripheral nervous system disorder or severe neuromuscular disease; (6) history of leukemia, severe chronic liver disease, solid organ transplantation, malignant tumor (because of their immunocompromised state and increased risk of infection); or (7) severe cardiac disease (to avoid provocation of hemodynamic instability).

### Measurements

Patients were ventilated with a SERVO-i ventilator (Maquet, Solna, Sweden). A nasogastric feeding tube (NeuroVent Research, Toronto, ON, Canada) with electrodes to measure EAdi and balloons to measure esophageal pressure (Pes) and gastric pressure (Pga) were inserted through the nose. After verifying the correct positioning of the esophageal balloon by an occlusion test with spontaneous breaths [13], and after accurate positioning was confirmed by EAdi signals on a dedicated window on the SERVO-i ventilator (according to the manufacturer’s recommendations), the nasogastric tube was secured. Flow and Paw measurements were acquired from the SERVO-i ventilator, and Pes and Pga levels were acquired via pressure transducers (NeuroVent Research).

Throughout the protocol, EAdi, Paw, flow, Pes and Pga waveforms were recorded by using a custom-made system (NeuroVent Research) for offline analysis. EAdi, Paw, flow and tidal volume (Vt) were acquired from the SERVO-i ventilator via the RS232 serial port at a sampling rate of 100 Hz. Pes and Pga were acquired at 2 KHz using via a DT 304 A/D board (data translation) and synchronized to the RS232 serial port signals at 100 Hz. Ptp was calculated as Paw − Pes.

### Study protocol

Patients were receiving intravenous analgesia with morphine at 1 to 2 mg/hr, and no sedative was used during the experimental period. Patients were ventilated in pressure support mode (set by the attending physician) for 30 minutes after the nasogastric tube insertion. Patients were then switched to NAVA, with the NAVA level initially set to 0 for 3 minutes. Positive end-expiratory pressure (PEEP) and fraction of inspired oxygen (FiO_2_) were set as prescribed by the attending physician. The NAVA level was then increased every 3 minutes in steps of 0.3 cmH_2_O/μV to a NAVA level of at least 3.0 cmH_2_O/μV. The upper pressure limit was set to 45 cmH_2_O in order not to interfere with the titration. At each NAVA level, one non-assisted breath was obtained by reducing the NAVA level to 0 (hereafter NAVA zero breath). PEEP was unchanged during these breaths. At every NAVA level, one inspiratory occlusion was performed to measure neuromechanical efficiency (NME), as described below.

### Offline signal processing

**Figure 1:**
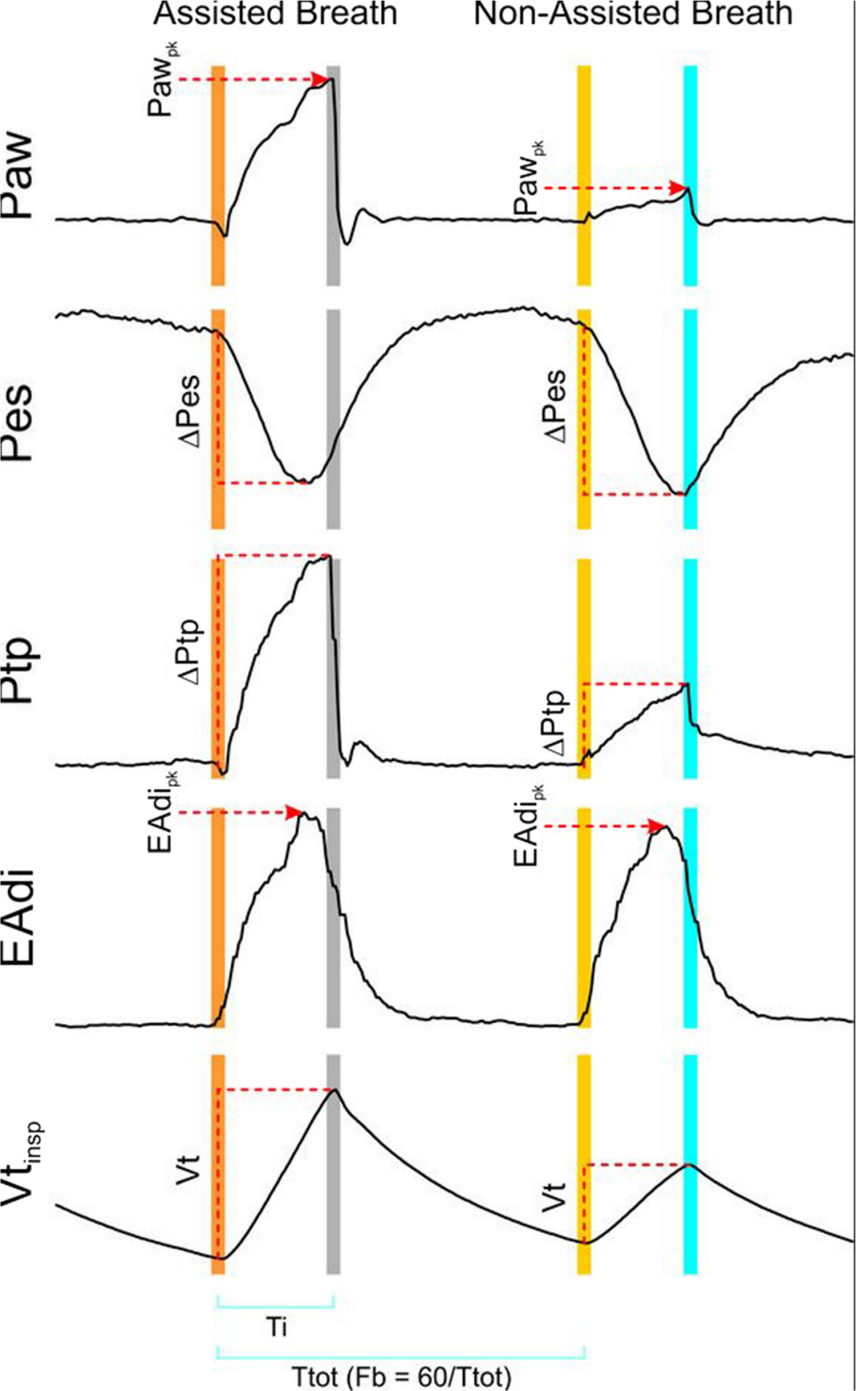
**Description of reference points used for calculation of respiratory variables.** Reference points are indicated for calculating peak airway pressure (Paw_pk_), change in esophageal pressure (Pes) from onset of electrical activity of the diaphragm (EAdi) to peak (nadir) (ΔPes), change in transpulmonary pressure (Ptp) from onset of EAdi to peak (ΔPtp), peak EAdi (EAdi_pk_) and inspiratory tidal volume (Vt_insp_). Neural inspiratory period (Ti) and total breath time (Ttot) are indicated at bottom. Breathing frequency (Fb) was calculated as 60/Ttot.

At each NAVA level, the last five breaths (prior to the NAVA zero breath) were analyzed. Figure 1 illustrates measurement points for signal processing. Neural inspiratory time (Ti) was defined as the time between the onset of increasing EAdi and the point where EAdi dropped to 70% of its peak. Although EAdi_pk_ could be suggested as the end of Ti, the EAdi drop to 70% of its peak was the off-cycling criterion for NAVA and thus allowed Ti to include detection of peak pressures and integration of inspiratory volume throughout the entire assist cycle (Figure 1). During each breath’s Ti period, EAdi_pk_) and peak airway pressure (Paw_pk_) were calculated. As well, ΔPes and ΔPtp between the onset of EAdi to their respective peak values were calculated. Note that the Pes signal has a negative trajectory and negative peak. Note also that peak values were obtained anytime during Ti and were not exactly matched in time.

Mean values for changes in EAdi and Pes were also calculated for each Ti period. Mean EAdi was obtained by integration during the Ti period and then divided by Ti. Mean Pes and Ptp were calculated the same way; however, values used during Ti integration were subtracted by the values measured at onset each of Ti. The mean and peak values for EAdi, ΔPes, Paw and ΔPtp were closely related (range of *R*^2^ = 0.94 to 0.97), and, for this reason, mean values are not reported. Of note, the mean values represented 60% to 70% of the peak values.

Breathing frequency (Fb) was calculated on a breath-by-breath basis as 60 divided by total breath time (that is, time between consecutive onsets of EAdi).

In order to quantify presence of paradoxical expiratory muscle relaxation during the neural inspiration, we calculated the change in Pga (ΔPga_paradox_) between onset of EAdi and the lowest value observed during Ti for the assisted breath(s) preceding the non-assisted breath.

NME was calculated as Paw_pk_/EAdi_pk_ during inspiratory occlusions at each NAVA level [14]. This index provides information about inspiratory muscle contractility and whether NME could be affected by the protocol (for example, by dynamic hyperinflation).

### Main analysis

PVBC was calculated as (Vt_insp_/EAdi_pk_)_no-assist_/(Vt_insp_/EAdi_pk_)_assist_. Another index, PVBC^2^, was calculated, simply by squaring PVBC [12]. Previous experimental work [12] showed that squaring PVBC was useful as a correction to improve its relationship to ΔPes/ΔPtp.

ΔPes/ΔPtp (sign was reversed to obtain positive index value) was used as a direct index to quantify the patient inspiratory muscle effort (ΔPes) contribution to total lung-distending pressure (ΔPtp) during assisted breaths [12]. As the assist (Paw) increases, the patient’s mechanical inspiratory effort (ΔPes) decreases (that is, becomes less negative), thus reducing its fraction relative to the total inspiratory effort ΔPtp (Ptp = Paw − Pes), indicating the relative contribution of patient inspiratory pressure and total inspiratory pressure.

In our previous study using the experimental animal model, both PVBC and PVBC^2^ were related to ΔPes/ΔPtp [12] during increasing NAVA levels. In the animal study, respiratory drive (and its response) was fairly uniform. However, in non-sedated humans, intrabreath variability is often higher. In addition, we studied a heterogeneous group with a wide range of EAdi values at zero NAVA level. For this reason, we evaluated methods of refining the accuracy and stability of the PVBC and PVBC^2^ indices as described in the subsections below.

#### Single versus averaged breaths

We calculated the PVBC and PVBC^2^ indices in two ways: (1) using the same method described previously [12], applying a single non-assisted breath (N1) compared with the previous single breath (referred to as ^N1^PVBC and ^N1^PVBC^2^); and (2) using PVBC and PVBC^2^ calculated with five averaged EAdi values from the five preceding breaths (“X5”) (referred to as ^X5^PBVC and ^X5^PBVC^2^).

#### Matching respiratory drive between assisted and non-assisted breaths

Respiratory drive can be interpreted from both EAdi_pk_ and Ti. As mentioned in the Introduction above, to calculate the PVBC-related indices, we assumed that either respiratory drive should be fairly similar for the unassisted and the assisted breaths or breaths could be corrected by using respiratory drive to “normalize” Vt_insp_ [12]. To evaluate the effect of “matching” the respiratory drive for unassisted and assisted breaths on the relationship between PVBC and ΔPes/ΔPtp, we tested different criteria and/or filters. We calculated the ratio between EAdi_pk_ with and without assist (EAdi_pk,no-assist_/EAdi_pk,assist_) as well as Ti with and without assist (Ti_no-assist_/Ti_assist_) at each NAVA level. Assuming that a ratio of 1 was a perfect match, we arbitrarily decided upon five ranges that successively reduced the degrees of freedom for either EAdi_pk,no-assist_/EAdi_pk,assist_ or Ti_no-assist_/Ti_assist_: 0.63 to 1.60, 0.67 to 1.50, 0.71 to 1.40, 0.77 to 1.30 and 0.83 to 1.20. Each range was selected to show the same relative changes above and below 1.0. We also tested if closely matched EAdi_pk_ values between non-assisted and assisted breaths could eliminate the need for EAdi normalization in the PVBC indices. For this purpose, we calculated PVBC simply as the ratio between Vt_insp_ for non-assisted to assisted breaths (Vt_insp,no-assist_/Vt_insp,assist_), subsequently referred to as PVBCβ (using same annotations as the PVBC).

### Statistical analysis

Statistical analysis was performed with SigmaPlot 12.5 (Systat Software, San Jose, CA, USA). Values in the text and figures are mean ± 95% confidence interval, unless otherwise indicated.

The relationship between different variables was tested with linear regression analysis. To identify the impact of averaging and breath matching on *R*^2^-values for the PVBC–ΔPes/ΔPtp relationship, one-way repeated-measures analysis of variance was performed. *Post hoc* comparison was performed by using the Student–Newman–Keuls test. A significant difference was defined as *P* <0.05. Bland-Altman analysis was used to study agreement between methods.

## Results

As shown in Table 1, 12 patients (3 males; age range, 38 to 82 years; predicted body weight range, 51 to 66.5 kg) were studied (9 patients with pneumonia, 2 patients with cardiogenic pulmonary edema and 1 patient with acute respiratory distress syndrome). All patients were receiving supplemental oxygen (FiO_2_ = 0.4 or 0.5), with oxygen saturation ranging from 95% to 100%.

**Table 1:** **Patient descriptions** ^**a**^

**Patient**	**Age, yr**	**Sex**	**APACHE II score**	**Predicted weight, kg**	**Cause of respiratory failure**	**PEEP, cmH**_**2**_ **O**	**FiO**_**2**_	**SpO**_**2**_
1	58	F	12	65	Cardiogenic pulmonary edema	8	0.4	95
2	60	F	22	65	Cardiogenic pulmonary edema	6	0.4	98
3	32	F	16	52	Pneumonia	8	0.4	95
4	82	M	32	70	ARDS, intestinal infection	4	0.4	99
5	72	F	30	52	Pneumonia	5	0.4	98
6	38	M	18	75	Pneumonia	8	0.5	95
7	69	F	24	60	Pneumonia	5	0.4	100
8	81	F	30	64	Pneumonia	7	0.4	98
9	57	F	20	50	Pneumonia	4	0.4	98
10	76	F	28	50	Pneumonia	5	0.4	100
11	56	M	16	67	Pneumonia	5	0.4	100
12	60	F	28	58	Pneumonia	5	0.4	96
Mean (SD)	61.8 (15.6)		23.0 (6.6)	60.7 (8.0)		5.8 (1.5)	0.41 (0.02)	98 (2)

^a^APACHE II, Acute Physiology and Chronic Health Evaluation II; FiO_2_, Fraction of inspired oxygen; PEEP, Positive end-expiratory pressure; SD, Standard deviation; SpO_2_, Pulse oxygen saturation.

All subjects reached a NAVA level of 3.0 cmH_2_O/μV.

Group mean values for ΔPga_paradox_, the largest magnitude of paradoxical expiratory muscle relaxations during Ti, were −0.08 (0.06) and −0.08 (0.05) cmH_2_O during the single and five assisted breaths (preceding the non-assisted breaths), respectively.

**Figure 2:**
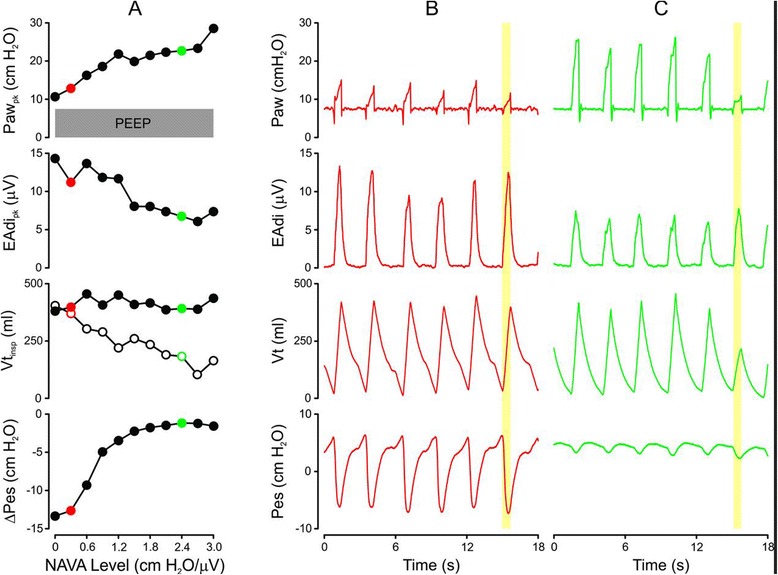
**Example of neurally adjusted ventilatory assist level titration in one patient. (A)** Effect of 11 increases of the neurally adjusted ventilatory assist (NAVA) level in steps of 0.3 cmH_2_O/μV (*x*-axis) on peak airway pressure (Paw_pk_), peak inspiratory electrical activity of the diaphragm (EAdi_pk_), tidal volume (Vt_insp_) during assisted (closed circles) and non-assisted (open circles) breaths and esophageal pressure peak inspiratory change from onset of EAdi (ΔPes). **(B)** and **(C)** Respective raw signals for Paw, EAdi, volume and Pes for five assisted and one unassisted breath (NAVA zero breath is indicated by yellow vertical bars) at a low NAVA level (**B**, red tracings), which relates to the red dot in **(A)**, and at high NAVA level (**C**, green tracings), which relates to the green dot in **(A)**.

Figure 2 exemplifies, in one patient, the effect of increasing NAVA level on Paw_pk_, EAdi_pk_, ΔPes and Vt_insp_ (Figure 2A). It also provides examples of time tracings for Paw, EAdi, Vt and Pes for five assisted breaths and one unassisted breath (yellow vertical shadow) at one low and one high NAVA level (Figure 2B and C) out of the total of eleven applied.

The NME did not change significantly from NAVA level zero (NME = 1.40 ± 0.63 cmH_2_O/μV) to the last titration step at NAVA level 3.0 cm H_2_O/μV (NME = 1.70 ± 0.86 cmH_2_O/μV).

**Figure 3:**
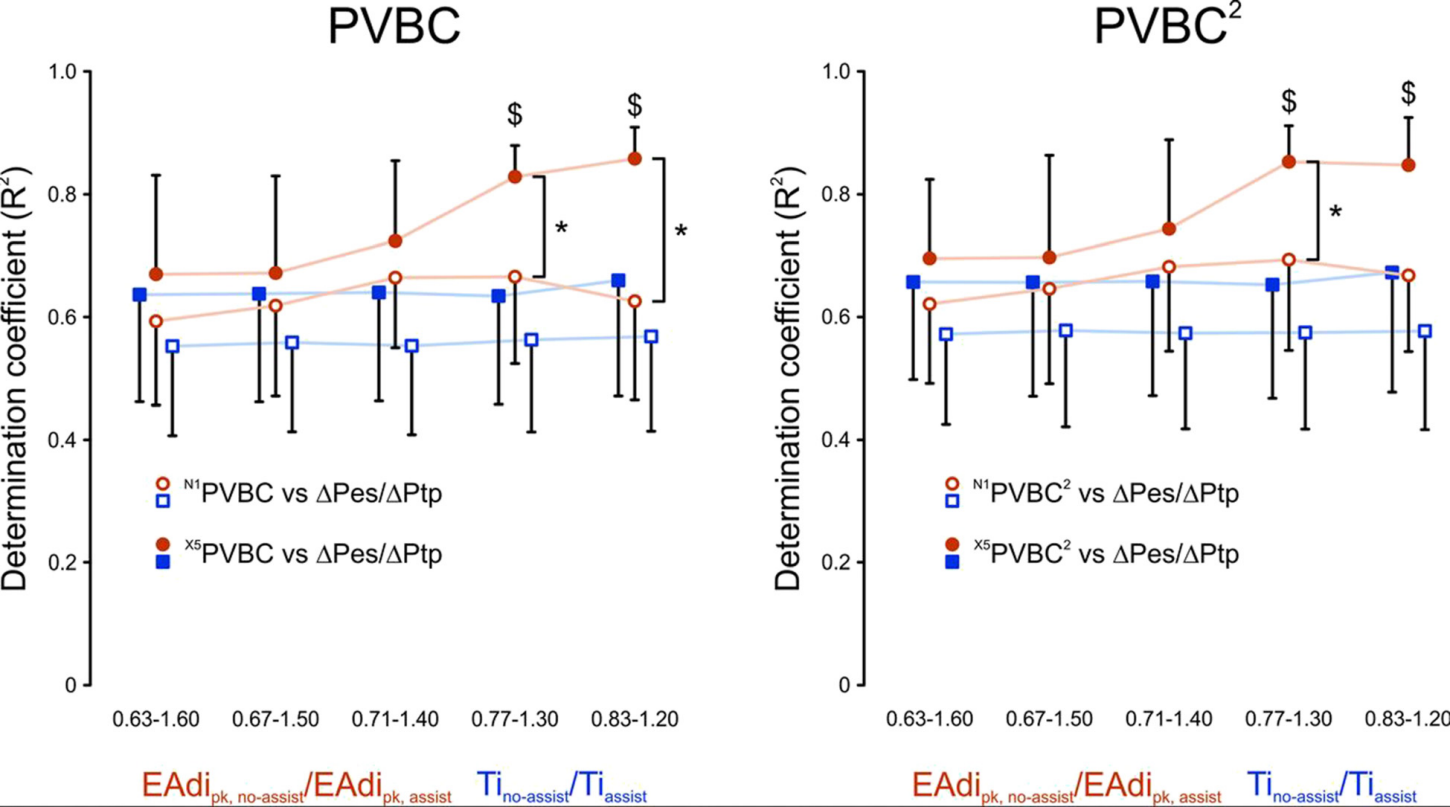
**Impact of breath averaging and breath matching on coefficient of determination between patient-ventilator breath contribution indices and ratio of inspiratory changes in esophageal pressure and transpulmonary pressure.** *Left*: Coefficient of determination (*R* ^2^) between patient-ventilator breath contribution (PVBC) and ratio of inspiratory changes in esophageal pressure and transpulmonary pressure (ΔPes/ΔPtp) (*y*-axis) is plotted against different matching criteria of increasingly strict inclusion levels (*x*-axis). Data are presented for PVBC indices when PVBC is calculated with one assisted breath (^N1^PVBC, open symbols) or with five assisted breaths averaged (^X5^PVBC, closed symbols). The determination coefficient was found to improve for ^X5^PVBC at matching levels for ratios of inspiratory peak electrical activity of the diaphragm (EAdi_pk_) with versus without assist (EAdi_pk,no-assist_/EAdi_pk,assist_) ranging from 0.77 to 1.30 ($*P* <0.05, orange closed symbols) and to become significantly higher than ^N1^PVBC (*P <0.05). Increasing the matching of the neural inspiratory time (Ti) between assisted and non-assisted breaths (Ti_no-assist_/Ti_assist_, blue symbols) did not result in any improvement in *R* ^2^. Values are presented as mean with 95% confidence interval. *Right*: Same as left graph, but for PVBC^2^. The *R* ^2^-value between PVBC^2^ and ΔPes/ΔPtp (*y*-axis) is plotted when PVBC^2^ is calculated with one assisted breath (^N1^PVBC^2^, open symbols) or with five assisted breaths averaged (^X5^PVBC^2^, closed symbols) and when different matching criteria are used of increasingly strict inclusion levels (*x*-axis). The determination coefficient was found to improve for ^X5^PVBC^2^ at matching levels for EAdi_pk,no-assist_/EAdi_pk,assist_ of 0.77 to 1.30 ($*P* <0.05, *P <0.05, orange closed symbols) and to become significantly higher than ^N1^PVBC^2^ (*P <0.05). Increasing the matching of the Ti between assisted and non-assisted breaths (Ti_no-assist_/Ti_assist_, blue symbols) did not result in any improvement in *R* ^2^. Values are presented as mean with 95% confidence interval.

Figure 3 shows how averaging five assisted breaths (closed symbols, “X5” notation) versus using one non-assisted breath (open symbols, “N1” notation) prior to the non-assisted breath influences the PVBC indices’ correlation to ΔPes/ΔPtp. It also shows the impact of respiratory drive “matching” for both EAdi_pk,no-assist_/EAdi_pk,assist_ (orange symbols) and Ti_no-assist_/Ti_assist_ (blue symbols) on the determination coefficients (*R*^2^, *y*-axes) between PVBC indices and ΔPes/ΔPtp. PVBC versus ΔPes/ΔPtp showed higher determination coefficients (*y*-axes) when calculated from the average of five preceding assisted breaths compared with when they were calculated with one assisted breath. Better matching of EAdi_pk,no-assist_/EAdi_pk,assist_ increased determination coefficients for PVBC indices with five breaths average, reaching significance with matching criteria (EAdi_pk,no-assist_/EAdi_pk,assist_) at 0.77 to 1.30.

**Figure 4:**
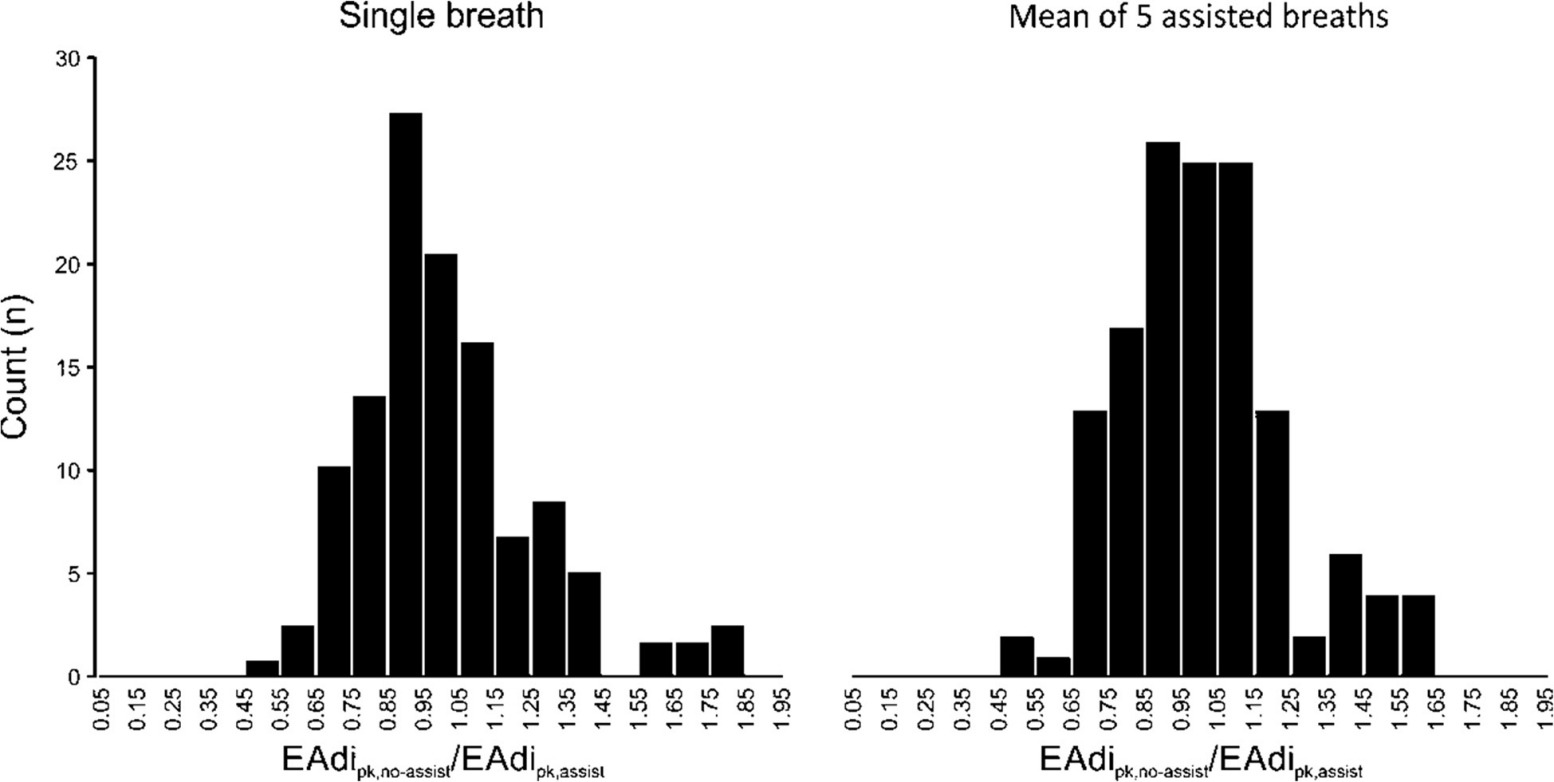
**Distribution of ratios between peak inspiratory electrical activity of the diaphragm without assist to peak inspiratory electrical activity of the diaphragm with assist.** Histograms showing distribution of all electrical activity of the diaphragm (EAdi) matching index values (EAdi_pk,no-assist_/EAdi_pk,assist_) when calculated using a single assisted breath (*left*) or using the average of five breaths preceding the non-assisted breath (*right*). Of all the breaths, 78% fell between 0.77 and 1.30.

Improved matching of Ti (*x*-axes in Figure 3) did not improve determination coefficients (*y*-axes in Figure 3) between PVBC indices and ΔPes/ΔPtp. We found that the EAdi_pk,no-assist_/EAdi_pk,assist_ of 0.77 to 1.30 provided the best combination of highest *R*^2^ values, lowest variability and least exclusion of data points not meeting matching criteria. In summary, this analysis showed the highest determination coefficients between the PVBC indices and ΔPes/ΔPtp when the PVBC was calculated using an average of five assisted breaths with EAdi_pk,no-assist_/EAdi_pk,assist_ within the 0.77 to 1.30 range. The distribution of EAdi_pk,no-assist_/EAdi_pk,assist_ for all patients and NAVA levels is presented in Figure 4.

Henceforth, results are reported only for patients with EAdi_pk,no-assist_/EAdi_pk,assist_ ratios between 0.77 and 1.30.

**Figure 5:**
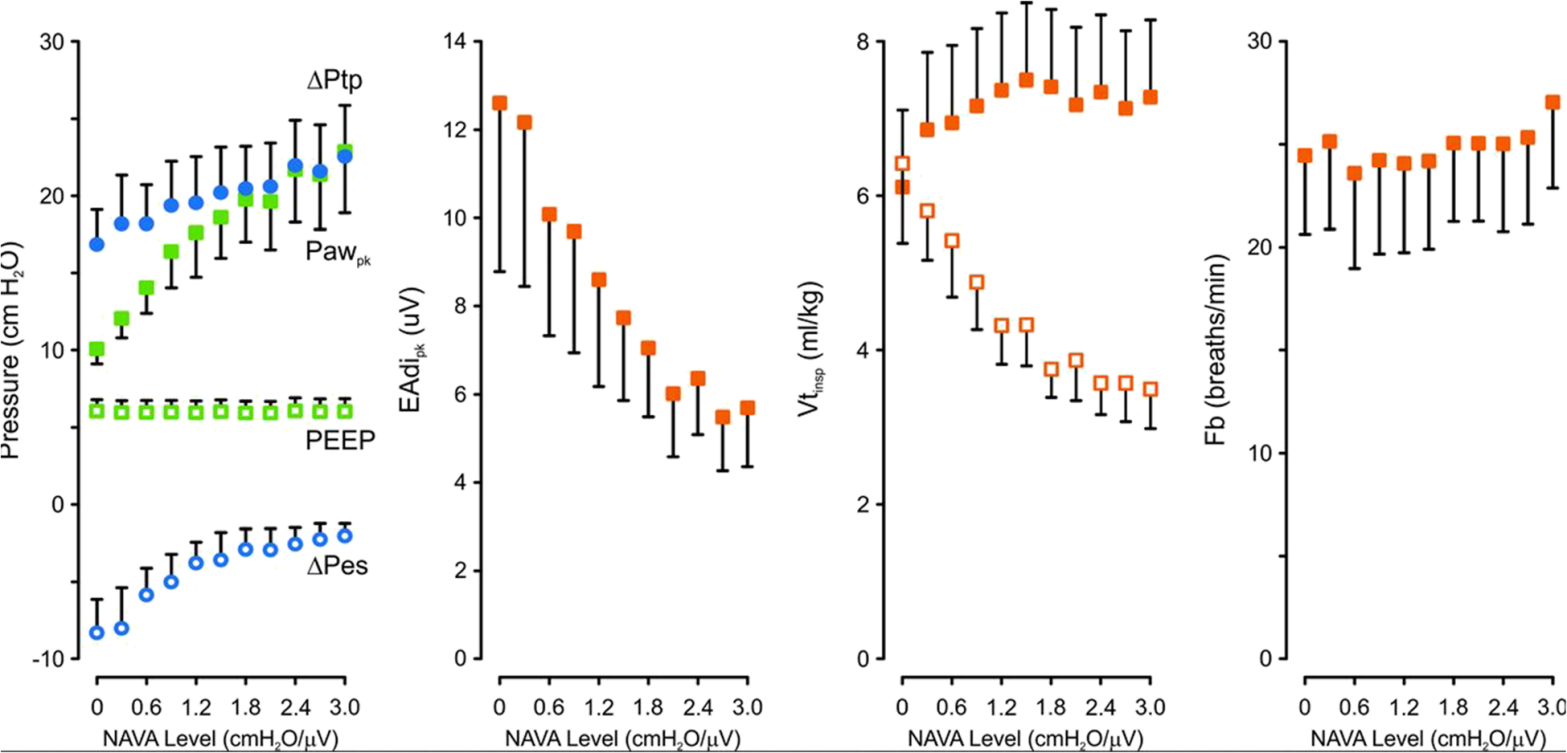
**Group mean values of measured variables during neurally adjusted ventilatory assist level titration.** *Leftmost panel*: Mean and 95% confidence interval values (*y*-axis) for peak inspiratory change in esophageal pressure from onset of electrical activity of the diaphragm (ΔPes, open blue circles), applied positive end-expiratory pressure (PEEP, open green squares), peak airway pressure (Paw_pk_, green solid squares), peak inspiratory change in transpulmonary pressure from onset of electrical activity of the diaphragm (ΔPtp, blue solid circles) above PEEP, as the neurally adjusted ventilatory assist (NAVA) level was increased from 0 to 3.0 cmH_2_O/μV (*x*-axis). *Next three panels to the right*: Mean and 95% confidence interval values plotted for peak electrical activity of the diaphragm (EAdi_pk_), inspiratory tidal volume (Vt_insp_) for assisted (closed symbols) and non-assisted breaths (open symbols) and breathing frequency (Fb) during increasing NAVA levels.

Figure 5 shows group mean values for ΔPes, Paw_pk_ (including PEEP), ΔPtp (including PEEP), PEEP, EAdi_pk_, Vt_insp_, and Fb for increasing NAVA levels. With progressive increases in NAVA level, there was a concomitant reduction in EAdi_pk_ until a point where the increase in Vt plateaued. Fb did not change for the titration of NAVA levels.

**Figure 6:**
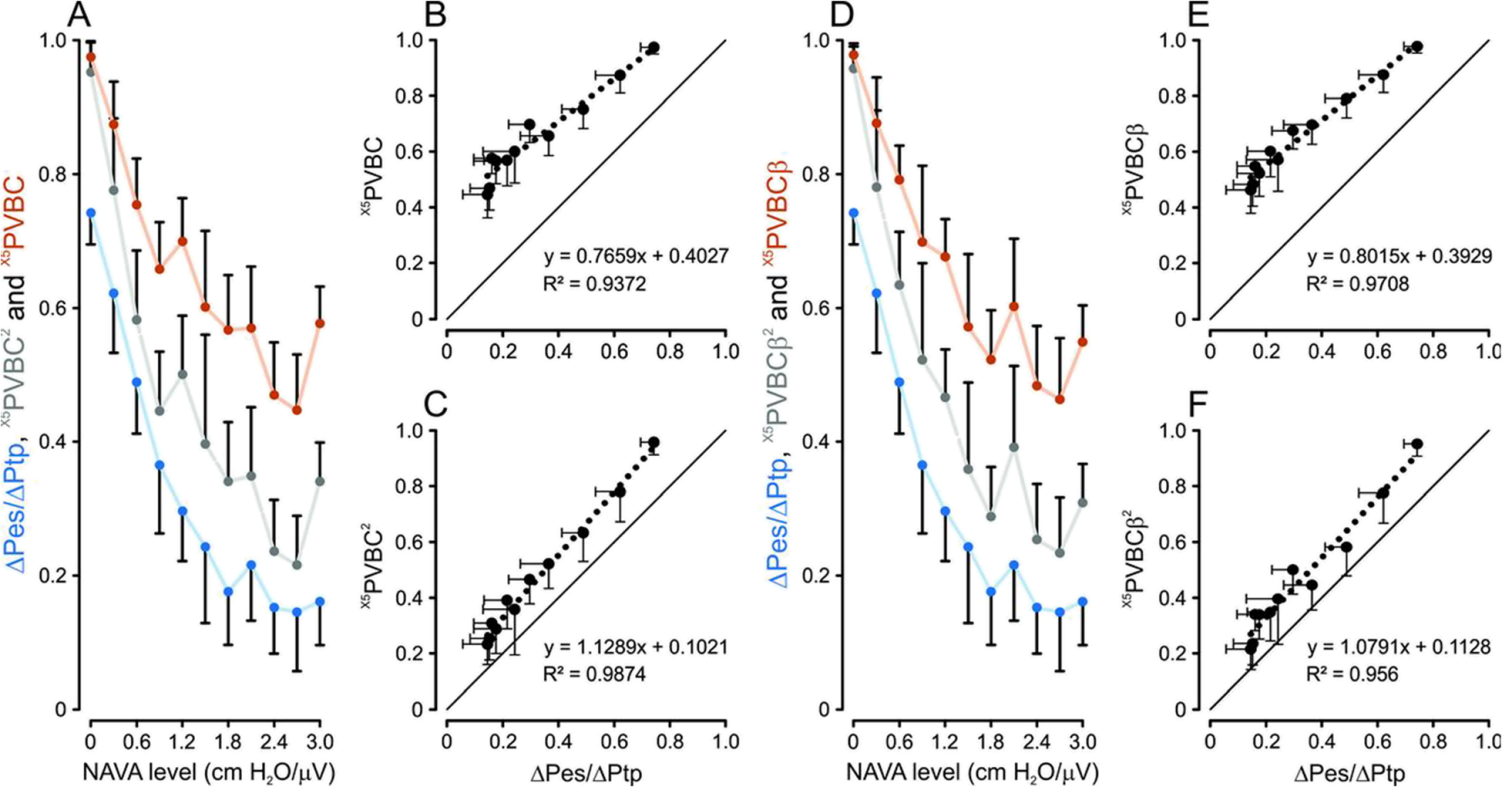
**Relationship between patient-ventilator breath contribution indices and ratio of inspiratory changes in esophageal and transpulmonary pressure indices with and without electrical activity of the diaphragm normalization. (A)** Group mean values and 95% confidence interval for indices of patient-ventilator breath contribution calculated from single non-assisted breath compared with five averaged electrical activity of the diaphragm (EADi) values from the five preceding breaths (^X5^PVBC), ^X5^PVBC^2^ and ratio of inspiratory changes in esophageal and transpulmonary pressure (ΔPes/ΔPtp) with increasing neurally adjusted ventilatory assist (NAVA) levels. **(B)** and **(C)** ^X5^PVBC and ^X5^PVBC^2^ plotted against ΔPes/ΔPtp, respectively. Note how ^X5^PVBC^2^ corrected the regression slope against ΔPes/ΔPtp. **(D)** Group mean values and 95% confidence intervals for ^X5^(PVBCβ), ^X5^(PVBCβ)^2^ and ΔPes/ΔPtp indices with increasing NAVA levels. **(E)** and **(F)** ^X5^(PVBCβ) and ^X5^(PVBCβ)^2^ plotted against ΔPes/ΔPtp, respectively. Note how ^X5^PVBCβ^2^ corrected the regression slope against ΔPes/ΔPtp. All values were calculated with the five-breath averaging technique and EAdi matching criteria (EAdi_pk,no-assist_/EAdi_pk,assist_) of 0.77 to 1.30.

Figure 6A illustrates the group mean values for ^X5^PVBC, ^X5^PVBC^2^ and ΔPes/ΔPtp with increasing NAVA levels. Figures 6B and C show ΔPes/ΔPtp (*x*-axes) plotted against ^X5^PVBC and ^X5^PVBC^2^ (*y*-axes), respectively. Figure 6D shows the same information as Figure 6A, but with PVBC calculated without normalizing Vt_insp_ by EAdi_pk_ (referred to as ^X5^PVBCβ and ^X5^PVBCβ^2^). The proportionality between ^X5^PVBCβ versus ΔPes/ΔPtp as well as ^X5^PVBCβ^2^ versus ΔPes/ΔPtp is demonstrated in Figure 6E and F.

As the NAVA level increased, ^X5^PVBC and ^X5^PVBC^2^ as well as ^X5^PVBCβ and ^X5^PVBCβ^2^ (panels A and D) decreased in a fashion similar to that of ΔPes/ΔPtp, showing less contribution by the patient. Squaring the indices significantly adjusted the regression coefficients and moved the intercepts for ^X5^PVBC^2^ (panel C) and ^X5^PVBCβ^2^ (panel F) closer to zero.

Table 2 shows group mean values for bias, standard deviation, lower and upper levels of agreement and precision of the Bland and Altman analysis for ^X5^PVBC, ^X5^PVBC^2^, ^X5^PVBCβ and ^X5^PVBCβ^2^ indices versus ΔPes/ΔPtp. Squared indices, ^X5^PVBC^2^ and ^X5^PVBCβ^2^, reduced bias (P <0.05), and better agreement for lower (P <0.05) and upper (P <0.05) levels of adjustment with regards to ΔPes/ΔPtp. All data in Figure 6 and Table 2 use the EAdi_pk_ ratio inclusion criteria of 0.77-1.30 between single non-assisted breath and mean of previous 5 breaths.

**Table 2:** **Bland-Altman analysis for** ^**X5**^ **PVBC,** ^**X5**^ **PVBC** ^**2**^ **,** ^**X5**^ **PVBCβ and** ^**X5**^ **PVBCβ** ^**2**^ **with reference to ΔPes/ΔPtp** ^**a**^

	^**X5**^ **PVBC**		^**X5**^ **PVBC** ^**2**^		^**X5**^ **PVBCβ**		^**X5**^ **PVBCβ** ^**2**^	
	**Mean**	**95% CI**	**Mean**	**95% CI**	**Mean**	**95% CI**	**Mean**	**95% CI**
Bias	0.34	0.05	0.16*	0.06	0.33	0.05	0.16*	0.06
SD	0.10	0.01	0.10	0.02	0.09	0.02	0.08	0.02
Lower LoA	0.16	0.07	−0.04*	0.09	0.16	0.07	−0.01*	0.08
Upper LoA	0.56	0.07	0.35*	0.04	0.50	0.06	0.32*	0.06
Precision	0.40	0.06	0.39	0.07	0.34	0.07	0.33	0.08

^a^CI, Confidence interval; LoA, Level of adjustment; ΔPes/∆Ptp, Ratio of inspiratory changes in esophageal and transpulmonary pressure; PVBCβ, Patient-ventilator breath contribution as ratio of inspiratory tidal volume for non-assisted to assisted breaths; ^X5^PBVC, Patient-ventilator breath contribution calculated from single non-assisted breath compared with five averaged electrical activity of the diaphragm values from the five preceding breaths; SD, Standard deviation. *P <0.05 versus ^X5^PVBC and ^X5^PVBCβ.

## Discussion

The results of the present study validate a clinical index that can be used to quantify the relative inspiratory effort of a patient during assisted ventilation. Comparison of Vt_insp_ corrected for neural inspiratory drive for a non-assisted breath (Vt_insp_/EAdi_pk_)_no-assist_ to that during an assisted breath (that is, (Vt_insp_/EAdi_pk_)_assist_) resulted in a PVBC index that closely reflected the ratio between patient (ΔPes) and total (patient + ventilator = ΔPtp) mechanical inspiratory effort. We also demonstrate that selection of non-assisted and assisted breaths with well-matched neural efforts improved the reliability of the PVBC index. Computation using an average of the five preceding assisted breaths, squaring PVBC and including only breaths with EAdi ratios for non-assisted and assisted breaths between 0.8 and 1.3 resulted in the most reliable PVBC (^X5^PVBC^2^) index. Using EAdi to match non-assisted and assisted breaths eliminated the need to correct for changes in neural respiratory drive and allowed computation of PVBC based on inspiratory volume alone.

The results of our clinical study of patients with ARF generally confirm those of the previous study in animals by Grasselli *et al*. [12]. We found that the coefficient of determination (*R*^2^) between PVBC indices and ΔPes/ΔPtp improved when neural inspiratory drive was matched between assisted and non-assisted breaths. Neural drive can be considered to have two major components: (1) neural breathing frequency (that is, the periodic repetition of neural activation, which is a temporal indicator of neural respiratory drive) and (2) amplitude (that is, the magnitude of neural respiratory drive). Our finding that matching assisted and non-assisted breaths for Ti had little effect on *R*^2^ between PVBC and ΔPes/ΔPtp suggests that temporal variability in neural drive had little influence on the accuracy of the PVBC index. However, EAdi_pk_ had a strong impact in improving the fit between PVBC and ΔPes/ΔPtp, suggesting that the magnitude of neural inspiratory effort is important for the accuracy of the PVBC index.

Transesophageal measurements of EAdi mainly represent motor unit activation for a portion of the crural diaphragm. Despite this, studies have demonstrated that the EAdi provides a good measure of the global diaphragm activation in healthy subjects [15] and mechanically ventilated patients with ARF [16]. Nonetheless, changes in respiratory muscle recruitment may cause alterations of chest wall configuration that would change NME between assisted and non-assisted breaths [15-17]. In the present study, the NME obtained during the occlusions was not affected by increasing NAVA levels.

The present study demonstrates a better fit to the regression line (higher *R*^2^ values) between PVBC and ΔPes/ΔPtp when an average of five assisted breaths was used compared with a single breath. We did not apply this approach for the non-assisted breaths because of clinical concerns about removing the assist for several breaths and the potential of altering respiratory muscle recruitment for the various breaths.

Grasselli *et al*. [12] showed that squaring PVBC values linearized the regression slope obtained with ΔPes/ΔPtp with changes in NAVA levels, and it also moved the intercept closer to zero. Our findings were similar, although they were not as evident. Squaring ^X5^PVBC altered the regression slope and moved the intercept closer to zero compared with ^X5^PVBC; however, there was no group mean increase of improved fit to the regression line between PVBC and ΔPes/ΔPtp as would be expected if curvilinearity had been removed. Yet again, this could be due to the wide heterogeneity in physiological and pathological factors between patients.

Our finding that a ratio of EAdi between assisted and non-assisted breaths of 0.78 to 1.30 produced equally good fit to ΔPes/ΔPtp when we calculated ^X5^PVBCβ^2^ from “only” the ratio of Vt_insp_ from unassisted to assisted breaths (that is, without normalizing to EAdi) confirms our initial hypothesis that when inspiratory effort is matched between assisted and non-assisted breaths, Vt_insp_ must change if synchronized assist is added or removed. Hence, the ratio of Vt_insp_ values for non-assisted and assisted breaths should be close to a value of 1 when no assist is being provided (the patient generates all the volume) and near zero when a breath is fully assumed by the ventilator.

### Critique of methods

The results given in the present study relate to peak values (that is, changes in amplitude in the signal between the onset of EAdi and the peak of a signal). Regarding Paw, one could argue that because NAVA does not have a plateau pressure, peak and mean values would provide different information. Regression analysis indicated extremely high correlation between the peak and mean values for Paw. Similarly, other variables, such as ΔPes, ΔPtp and EAdi_pk_, showed extremely strong relationships to their respective mean values. Given that pressures and EAdi are presented as peak values on the ventilator, we preferred to present results that had the closest relationship to clinical implementation.

In spontaneously breathing patients recovering from acute respiratory failure, expiratory muscle contraction during expiration followed by expiratory muscle relaxation during the ensuing inspiration may invalidate and amplify the decrease in esophageal pressure [18]. Such an overestimation of ΔPes can be detected by an end-expiratory decrease in Pga causing a concomitant decrease in Pes that is not related to inspiratory muscle effort [19]. In the present study, we used onset of EAdi as the starting point to calculate ΔPes, which ensured that diaphragm contraction had started and eliminated the possibility for preinspiratory overestimation of the ΔPes. Our quantification of paradoxical expiratory muscle relaxation during neural inspiration as ΔPga_paradox_, calculated as the change in Pga between onset of EAdi and the lowest value observed during Ti for the assisted breath(s) preceding the non-assisted breath, was miniscule. The latter shows that end-expiratory expiratory muscle relaxation was not an important contributor to our results.

As depicted in Figure 6, both ^X5^PVBC^2^ and ^X5^PVBCβ^2^ showed values about 0.1 relative units higher than ΔPes/ΔPtp. One reason for higher PVBC indices was that ΔPes decreases more than EAdi during assisted breaths. In fact, EAdi_pk_ does not reach zero when ΔPes indicates 100% unloading [20]. This means that for a given EAdi_pk_, non-assisted breaths can generate more Vt_insp_ than during assisted breaths [12]. Moreover, at a NAVA level of zero, the assist is not a true “zero assist,” because the SERVO-i ventilator still delivers a pressure of 2 cmH_2_O. These factors would act to shift PVBC indices upward.

Relaxation of the chest wall or expiratory muscle recruitment prior to EAdi off-cycling opens the expiratory valve during assisted breaths. This is due to the increase in Paw_pk_ by 3 cmH_2_O above target, causing a pressure off-cycling algorithm to be activated. If this happens, the Paw_pk_ is somewhat (3 cmH_2_O) higher than expected and the ΔPes/ΔPtp ratio would be lower than expected, especially at the lowest NAVA levels. Another reason that the ΔPes/ΔPtp ratio was less than 1.0 at the lowest NAVA level could be that Paw was lost because the endotracheal tube resistance led to overestimation of the Paw used to calculate Ptp.

Our finding of a 16% bias with the Bland-Altman analysis probably relates to a combination of overestimating PVBC as well as underestimating ΔPes/ΔPtp.

A limitation of this study’s design is that we did only one “PVBC maneuver” (NAVA zero breath) per NAVA level. Consequently, if the breath without assist failed to meet the inclusion criteria for EAdi_pk_ or Ti matching, we would lose the PVBC calculation for that NAVA level. If applied as an automatic function for use in the clinic, the non-assisted breaths could be repeated intermittently to increase the likelihood of obtaining acceptable and reproducible PVBC values.

Breathing patterns are affected by many factors, and stable activation and rhythm require a low level of disturbance. The protocol of the present study was performed by manual interventions by the investigators at the bedside, which could have distracted the patients compared with a system with built-in algorithms for non-assisted breaths and occlusions.

Three minutes at each NAVA level could be considered short; however, our data indicate that this was sufficient to produce clear reductions in both ΔPes and EAdi_pk_ for each NAVA level. Viale and colleagues showed that about five breaths were sufficient for patients to adapt and adjust to the new assist level during pressure support ventilation [21]. In the present study, we therefore focused on relatively quick unloading rather than on the effects of CO_2_ and pH on respiratory drive. To the contrary, our data showing that 78% of the breaths fell within the 0.77 to 1.30 matching criteria (one breath to the next) strongly suggest that the first unassisted breath is affected little by removal of ventilatory assist.

### Clinical relevance

In previous clinical studies, researchers have reported, on average, EAdi_pk_ values of about 10 μV when the NAVA level was adjusted to match Paw_pk_ during pressure support, providing clinically acceptable Vt levels [5,22,23]. (Note that although the absolute value of EAdi can be influenced by anatomical differences, this average value is representative of published data obtained from over 560 patients.) According to the results of the present study, this corresponds to average ^X5^PVBC^2^ and ^X5^PVBCβ^2^ indices of about 0.5 (that is, indicating the patient’s contribution is about half the total effort). It is interesting to observe that at an EAdi of 10 μV, the ΔPes was about −5 cmH_2_O, which could be considered a normal workload. As demonstrated previously during synchronized assist with NAVA, breathing frequency provided little information about acute changes in respiratory load and drive. As depicted in Figure 5, the initial increase in Paw and ΔPtp decreased at NAVA levels of 1.5 to 1.8. It has been hypothesized that the onset of this plateau indicates that the patient is “satisfied” by the assist [24]. In the present study, this occurred at an average EAdi of 7 to 8 μV, with ^X5^PVBC^2^ and ^X5^PVBCβ^2^ indices of about 0.3 to 0.4 (that is, when the patient’s contribution was about one-third of total effort). Although the highest precision of 0.3 (Bland-Altman analysis) obtained with ^X5^PVBCβ^2^ could be considered somewhat low, targeting ^X5^PVBCβ^2^ of 0.5 while monitoring EAdi with a target 10 μV would likely improve the ability to control patient unloading. This type of monitoring would have a direct impact on how to set the ventilator’s assist and, perhaps more important, would provide clinical studies aimed at ensuring avoidance of overassist and optimizing assist for faster weaning. In the present study, PVBC analysis was done by neurally synchronized and proportional assist. Principally, the comparison of Vt_insp_ for assisted and non-assisted breaths of similar EAdi amplitude should also work with pressure support ventilation; however, future studies are required.

## Conclusions

The present study demonstrates a clinical index used to quantify the relative inspiratory effort of a patient during assisted ventilation. Computation of PVBC as a ratio of Vt values from one non-assisted breath to the average of five preceding assisted breaths, squaring PVBC and including only breaths with EAdi_pk_ whose ratio between non-assisted and assisted breaths is within the range of 0.77 to 1.30 results in a clinically useful PVBC index.

## Key messages

- The ratio of inspired tidal volume (Vt_insp_) and inspiratory peak electrical activity of the diaphragm (EAdi_pk_) between non-assisted and assisted breaths, defined as patient and ventilator breath contributions (PVBCs), can be used to predict the relative contribution of the inspiratory muscles versus that of the ventilator during neurally adjusted ventilatory assist (NAVA).
- Computation of PVBC as a ratio of tidal volumes from one non-assisted breath to the average of five preceding assisted breaths, squaring PVBC and including only breaths with EAdi_pk_ whose ratio between non-assisted and assisted breaths is within the range of 0.77 to 1.30 resulted in a clinically useful PVBC index.

## Abbreviations

APACHE II: Acute Physiology and Chronic Health Evaluation II, ARF, Acute respiratory failure
CI: Confidence interval
EAdi: Electrical activity of the diaphragm
EAdi_pk_: Peak inspiratory electrical activity of the diaphragm
Fb: Breathing frequency
FiO_2_: Fraction of inspired oxygen
ICU: Intensive care unit
^N1^PVBC: Patient-ventilator breath contribution calculated from single non-assisted breath compared with the previous single breath
NAVA: Neurally adjusted ventilatory assist
NME: Neuromechanical efficiency
Paw: Airway pressure
Paw_pk_: Peak airway pressure
PEEP: Positive end-expiratory pressure
Pes: Esophageal pressure
ΔPes: Peak inspiratory change in esophageal pressure from onset of electrical activity of the diaphragm
Pga: Gastric pressure
Ptp: Transpulmonary pressure
∆Ptp: Peak inspiratory change in transpulmonary pressure from onset of electrical activity of the diaphragm
PVBC: Patient-ventilator breath contribution
SD: Standard deviation
SpO_2_: Pulse oxygen saturation
Ti: Neural inspiratory period
Ttot: Total breath time
Vt_insp_: Inspired tidal volume
^X5^PBVC: Patient-ventilator breath contribution calculated from single non-assisted breath compared with five averaged electrical activity of the diaphragm values from the five preceding breaths
